# What is the NHS Safety Thermometer?

**DOI:** 10.1258/cr.2012.012038

**Published:** 2012-09

**Authors:** Maxine Power, Kevin Stewart, Ailsa Brotherton

## Abstract

The English National Health Service (NHS) announced a new programme to incentivize use of the NHS Safety Thermometer (NHS ST) in the NHS Operating Framework for 2012/13. For the first time, the NHS is using the Commissioning for Quality and Innovation (CQUIN) scheme, a contract lever, to incentivize ALL providers of NHS care to measure four common complications (harms) using the NHS ST in a proactive way on one day per month. This national CQUIN scheme provides financial reward for the collection of baseline data with a view to incentivizing the achievement of improvement goals in later years. In this paper, we describe the rationale for this large-scale data collection, the purpose of the instrument and its potential contribution to our current understanding of patient safety. It is not a comprehensive description of the method or preliminary data. This will be published separately. The focus of the NHS ST on pressure ulcers, falls, catheters and urine infection and venous thromboembolism is broadly applicable to patients across all healthcare settings, but is specifically pertinent to older people who, experiencing more healthcare intervention, are at risk of not one but multiple harms. In this paper, we also describe an innovative patient-level composite measure of the absence of harm from the four identified, termed as “harmfreecare” which is unique to the NHS ST and is under development to raise standards for patient safety.

## Introduction

In 2012, the English National Health Service (NHS) embarked on a national incentive scheme under the Commissioning for Quality and Innovation scheme (CQUIN)^1^ to incentivize providers of NHS care (excluding patients under 65s in mental health units and paediatric patients) to take a snapshot measure of four common harms (pressure ulcers, falls, urinary infection [in patients with catheters] and venous thromboembolism [VTE]) on all patients being treated in NHS care on a predetermined date each month using the NHS Safety Thermometer (NHS ST).^2^ This move to take the national safety “temperature” on four common harms across all healthcare care settings is an ambitious programme that will result in an estimated 750 000 patients per quarter screened for harm. This paper summarizes the key learning from the development or “pilot” period (up to September 2011), prework period (October 2011–March 2012) and Quarter 1 of 2012/13 ahead of implementation of the CQUIN scheme in Quarter 2.

In the NHS, services are currently contracted from a wide range of providers including hospitals (providing acute NHS care to inpatients) and community providers (providing NHS care in patients' homes, nursing homes and community hospitals) by primary care trusts (commissioners). This commissioning responsibility will soon be transferred to newly established clinical commissioning groups. Acute care and community providers are commissioned using a standard national contract. In addition to normal “baseline” payments to providers for care, the CQUIN framework uses financial levers to incentivize providers to achieve certain quality goals.^3^ The national NHS ST CQUIN in 2012/13 incentivizes organizations to establish measurement systems and submit data collected using those systems according to the definitions and criteria set out in the NHS ST. Where providers achieve a CQUIN “goal”, in this case monthly surveying of all eligible patients in NHS-funded care using the NHS ST, they can earn an additional payment set as a proportion of the actual outturn value of the provider contract.

There are four national CQUIN goals (VTE risk assessment, dementia diagnosis, patient experience and the NHS ST), which should be included in all relevant contracts. Providers and commissioners also agree local schemes. For 2012/13, the Department of Health set the total amount that providers could earn via CQUIN as 2.5% on top of actual outturn value. The national goals have to represent around a fifth of the total value of a contract's CQUIN schemes. The rest should be made up of locally agreed schemes. This suggests a value for the NHS ST CQUIN of 0.125% of actual outturn value if all four national CQUIN schemes apply, or a greater percentage if fewer of the national CQUINs apply.

The informal reaction from frontline NHS services to the Department of Health initiative has been mixed.^4^ On one hand, some organizations are welcoming reward for activities that they perceive to improve the quality and safety of care. On the other, some see the incentive scheme as unnecessary, unwarranted bureaucracy and a potential source of “gaming”, with organizations potentially doing what is needed to meet the CQUIN goals without necessarily changing culture or practice. In this paper, we describe the rationale for this large-scale data collection, the purpose of the NHS ST instrument and its contribution to our current understanding of patient safety.

### Rationale

Despite numerous national policy^5–7^ and patient safety improvement programmes,^8^ with the exception of specific measures of hospital-acquired infection (Methicillin-resistant Staphylococcus aureus [MRSA] bacteraemia and *Clostridium difficile* diarrhoea),^9^ there is limited evidence of measureable improvements in hospital inpatient safety at a national level in England in the last decade.^10^ Inability to detect changes in safety is not specific to English healthcare.^11,12^ All healthcare systems are limited by current approaches to safety measurement, which demonstrate limitations in method if applied at scale, specifically with respect to reliability, consumption of time and ability to detect change over time (Box 1).

Box 1 **Measuring harm in healthcare: Patient Safety professionals and researchers use a standardized classification of adverse events. For research purposes most include any form of untoward incident which results in prolongation of the patient's hospitalization or has more serious implications (like long-term disability or death). They do not usually report harms which require intervention but do not prolong hospital stay, or other temporary harms (like minor drug administration errors). Harm from adverse events can be detected and measured by a number of methods including incident reports, structured case note review, analysis of routinely collected administrative data and prevalence surveys and audits. All have their strengths and limitations.**

- *Incident reports (also known as adverse events [AEs] and serious untoward events [SUIs])* are valuable for highlighting trends and patterns which might not be apparent at local level, but they have limited value in measuring harm. It is well recognized in all healthcare systems that incident reporting detects only 10–32% of adverse events or less. Organizational reporting culture is influenced by a number of complex factors which make it difficult to draw conclusions about frequency of events from incident reports.
- *Case note review (audit)* based on a standardized instrument (like the IHI Global Trigger Tool) probably gives the most complete picture of overall harm and is preferred by most researchers. It has well-recognized limitations; some conditions are not well recorded in medical case-notes and poor record keeping in general can distort results. It is resource intensive and requires significant infrastructure and personnel making its practical application difficult. Since it is a retrospective review there is limited opportunity for immediate intervention.
- *Administrative data* (like Hospital Episode Statistics data in England) are heavily dependent upon the quality of clinical coding. A recent report from the NHS Information Centre and the Academy of Medical Royal Colleges highlights the current poor quality of routinely collected administrative data in the UK which is attributed to lack of clinical involvement in coding. In the USA, where clinical coding is probably better, there are also well-recognized problems and this approach is generally not the preferred method for measuring harm.

In 2008, Vincent et al.^10^ concluded that “a lack of reliable information on safety and quality of care is hindering improvement in safety across the world”, describing increasing bias towards voluntary reporting of patient safety incidents, including adverse events and serious untoward incidents. There is no doubt that these reporting systems are a vital component of a learning healthcare system, providing warning and communication within and between organisations.^13^ However, they do not comprehensively measure harm. Recent Department of Health estimates have put the level of reporting to the NHS National Reporting and Learning Service of patient safety incidents leading to harm in inpatient care at 30% or less of the actual number of incidents of harm.^14^

Given the strength of the recommendations by Vincent et al., a shift in policy direction to include “a move away from unsystematic voluntary reporting towards systematic measurement” seems logical and inevitable. Vincent concludes that unless serious efforts are made to develop reliable indices of safety, we will still be unable to answer the question “are patients any safer in our care?” in 5 years’ time.

## Method

### The purpose and development of the NHS ST

**Figure 1:**
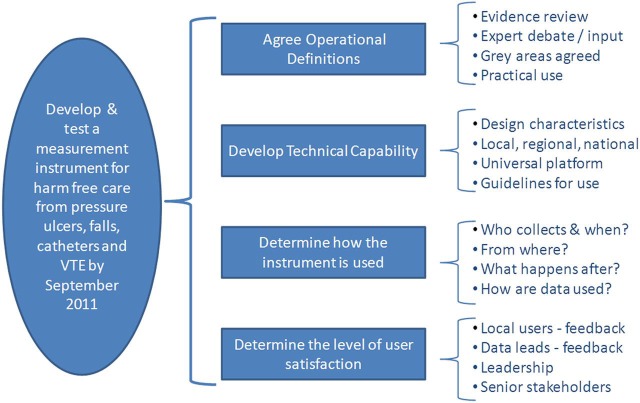
The schematic (driver diagram) illustrates the four key work programmes coordinated by the steering group during the development of the NHS ST. Each primary driver (in the dark blue box) had a work programme and carried out cycles of development, testing and adaptation during the development of the instrument

The NHS ST instrument has been designed to be used by frontline healthcare professionals to take a snapshot measure of pressure ulcers, harm from falls, urinary infection in patients with catheters and VTE, which can be aggregated to give whole organization, regional and national data using a simple merge function. It is called the NHS ST because it takes only minimum set of data that help to signal where individuals, teams and organizations might need to focus more detailed measurement, training and improvement. Users are asked to use the operational definitions in the tool, to gather information from clinical records, to examine the patient and to ask the patient simple questions as part of a routine activity (for example, a ward round or handover). Where the patient is unable to answer the question reliably, the primary carer is asked to provide the information. They are asked to do this for all patients receiving NHS-funded care (unless excluded) on one day per month, which provides a point estimate. The survey includes patients in hospital, care homes, community settings and their own homes. The sample includes patients receiving NHS-funded care on the day of the survey. The driver diagram for the development and testing of the NHS Safety Thermometer is presented in Figure 1.

The NHS ST was developed by organizations and individuals participating in the (QIPP). (The Quality, Innovation, Productivity and Prevention programme is a large-scale transformational programme for the NHS, involving all NHS staff, clinicians, patients and the voluntary sector. It is designed to improve the quality of care the NHS delivers while making up to £20 billion of efficiency savings by 2014–2015, which will be reinvested in frontline care.) “Safe Care” national programme as a response to the challenge of measuring harm in healthcare at a national level. The testing began in September 2010, following a development process for the operational definitions conducted from the Chief Nurse's office of the Department of Health. During the development process (2010–2011) a total of 161 organizations used the survey instrument. Of the patients surveyed, 80% were being cared for in acute hospital inpatient providers (all types), 10% were in their own home (and were surveyed by providers of peripatetic services, for example, district nursing or community rehabilitation), 2% were in a community hospital setting and 8% were in other settings, such as nursing homes. Overall, 46 284 entries were made into the survey (September 2010 to September 2011). Feedback mechanisms were established to gather intelligence on variations in implementation and user satisfaction including survey questionnaire, senior leader meetings and regional events. The survey instrument and method were modified throughout 2011 based on iterative feedback. Details of operational definitions (for each measure), sampling (including inclusions and exclusions), technical specification, guidance on collection and interpretation are available from the Department of Health,^15^ NHS Information Centre^16^ and “Harmfreecare” websites.^17^ In summary, the final feedback indicated that the instrument is intuitive, accessible and able to be used in less than 10 minutes per patient by frontline healthcare professionals with minimal additional training. These are essential qualities for an instrument that is to be applied to millions of patients. It can be used wherever the patient is located (home, community or hospital setting) again, essential for a healthcare system moving care closer to home. The instrument has a user interface that allows immediate review of data tables (for data verification) and graphical display for data over time (in simple line charts), with easy reference to peer groups and the national data. The data collected are easy to aggregate to show results at the ward/team, region or national level through the integral “merge” function which allows the collation of data at the press of a button.

## Results

**Figure 2:**
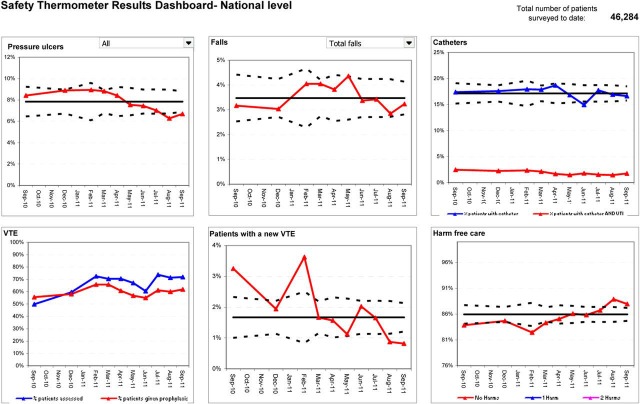
Excerpt from Implementing the NHS Safety Thermometer CQUIN, which displays monthly data over time (for 12 months from September 2010) for eight measures in the safety thermometer. Each data point represents the mean of all data submitted in month

Charts of data collected for 12 months (from September 2010) in Figure 2 show the data stabilizing towards the last 3 months of the pilot for all but one measure (VTE treatment). The final quarter (July–September 2011) show the prevalence of pressure ulcers was 6.7%, 3.2% of patients had fallen in the last 72 hours; 17% of patients had a catheter in situ; and 1.5% of patients had a catheter and were receiving treatment for a urinary tract infection. VTE risk assessment (in all patients surveyed) and administration of VTE prophylaxis (concordant with NICE guidance^18^) in those at high risk were recorded at 72% and 61%, respectively, by the end of the pilot. The proportion of patients being treated for a newly diagnosed VTE was inconsistent, stabilizing at 0.9%. The harm-free care composite indicator (indicating the absence of harm from any of the four outcomes in the thermometer) was achieved in 88% of patients.

While these data require further review and clarification, a review of available literature shows a promising comparison with small-scale epidemiological studies, systematic reviews of harm in healthcare^19^ and national audits, in particular with respect to pressure ulcers and catheter use (Box 2).

Box 2 **Review of literature on the four harms in the NHS ST**

*Pressure ulcers*

Pressure ulcer prevalence may be underestimated by traditional patient safety research. Routinely collected administrative data are also likely to significantly underestimate their prevalence as highlighted in a recent Welsh survey.^20^ In two patient safety studies from the US pressure ulcer prevalence was 6% and 7%, respectively^12,21^ and a Belgian survey of nearly 20 000 patients found grades 2–4 pressure ulcers in 7% of patients.^22^ In the UK, grades 2–4 pressure ulcers were present in 11% of patients in Welsh orthopaedic units and 17% of those in community hospitals.^23,24^

*Falls in care settings*

Accidental falls are the most commonly reported safety incident accounting for 200 000 reports to the National Patient Safety Agency (NPSA) in 2005/6, although this is almost certainly a significant underestimate. It has been estimated that an average ward will have about 10 falls per month of which 30% cause some harm and 1–5% lead to serious injury.^25,26^ Falls are associated with old age, confusion, multiple medications, impaired balance and acute illness in older patients.^27^

*Urinary tract infection in patients with catheters*

Most surveys show that between 15% and 20% of general hospital inpatients are catheterized. The risk of developing infection when a patient is catheterized is 3–7% per day and there is a clear association between length of catheterization and risk of infection. The most effective strategies for reducing infection are avoiding catheterization and limiting its duration.^28,29^

*Venous thromboembolism*

VTE causes 25 000 deaths per year in the UK. It is unclear how many of these are preventable but 50% of patients who develop a VTE have been hospitalized in the previous two months.^30^ A UK survey found that around 70% of “at risk” patients did not receive appropriate prophylaxis. There are contractual requirements for providers to risk assess all patients and provide prophylaxis where indicated.^31^

## Discussion

The NHS ST is engineered to focus attention of healthcare providers on a small number of key outcomes (harms), in a large number of patients, across the healthcare system, in a time efficient way. As stated, it offers advantage over other methods. It also has limitations. The intention of this paper is not to offer a detailed methodological description of the technical development of the NHS ST instrument. However, some technical issues relating to operational definitions remain a challenge and warrant consideration. Despite intent to focus on outcomes, our expert advisory groups were unable to definitively agree an outcome for catheter associated urinary tract infection or VTE which could be used across healthcare settings. VTE risk assessment, the appropriate administration of VTE prophylaxis in high-risk patients and the presence of a urinary catheter are three measures of process. Clinical treatment of a urine infection and commencement of VTE treatment are only a proxy for the actual outcome.

In much the same way as an increased temperature signals a need for further observations of a patient's physiological status, data collected using the NHS ST offer a gateway for focus that needs to be exploited by the relevant specialty groups for education and improvement. The approach is built on the premise that healthcare professionals, once alerted to harm, will understand that detailed scrutiny of underlying systems of care is required to drive changes in outcome. In other words, observed harm outcomes in the NHS ST are a “call to action” for increased attention to our harms, not an end in themselves. These assumptions are implicit in our theory of change. Their application is doubtlessly fragile and significant leadership, skill and culture change are required to capitalize on the increased awareness generated by the monthly collection. In those organizations where the implementation is being undertaken as a “must do” financial imperative, the risk of the “call to action” being lost is no doubt significant.

The method of data generation was developed during the testing period and a variety of tests of change resulted in agreement that a point estimate method should be used. The recommended method is an “opportunity sample” of all patients in NHS care on one predetermined day per month which, over time are used to represent the whole population.^32^ While this approach is statistically valid and affords significant advantage in reducing the “time spend” on measurement, it is counter-cultural in the National Health Service where significant importance is attached to methods derived from enumerative research and audit that require every patient to be reviewed.^33,34^ It is true that a point estimate measure (the occurrence of a given condition at a specific point in time) is more susceptible to variation depending on case-mix, seasonality and simple random variation. Specific charting (using run and control charts) and the application of rules that signal special cause variation are necessary to determine whether change has occurred.^35^ The application of special cause rules, while standard in engineering, is still relatively novel in healthcare and requires at least 10–12 data points before interpretations can be made.^36^ This time interval between the collection of data and the ability to make meaningful interpretations is challenging. In particular, the tendency to over- or under-react to changes from month to month is a legacy from our success in performance improvement in infection prevention and control, which again requires significant education, leadership and intelligence in data use.

In order to maximize the potential of the NHS ST, a mindset shift from data for comparison towards data for improvement over time is necessary. In looking at data in this way we accept that not all harm is avoidable but that improvements over time can yield improvements that are beyond our expectations. It is incorrect to assume that this approach is inferior. Measurement for improvement needs to have the same methodological vigilance as measures of performance.^37^ In particular, it is reliant on establishment of robust data collection systems to deliver an accurate baseline. Organizations that see exceptionally poor, good or variable performance are advised to scrutinize their collection methods carefully and review their data alongside other sources of data. Inconsistencies in data collection method, variable interpretation of operational definitions and failure to introduce local data quality checks are potential pitfalls for the novice. Indeed our understanding of previous attempts to establish data collections systems for central line infection in the Matching Michigan programme, signal strongly that aspects of the establishment process for collection matter greatly in the long-term application, utility and validity of the data.^38^

### Harm-free care and the elderly patient

Our available approaches to measure harm focus on the harm rather than the patient, e.g. the number of falls or the number of patients with infection, which conveniently align with our organization of clinical services, e.g. tissue viability services review pressure ulcer data, falls specialists review falls data. Clinically, it is well known that patients who suffer one harm have a high probability of developing another and may indeed have two or more.^12^ It is these patients for whom the burden, dependency and cost of suffering is greatest. It is these patients who are frail, elderly and most vulnerable. Yet rarely do we measure holistically.

**Figure 3:**
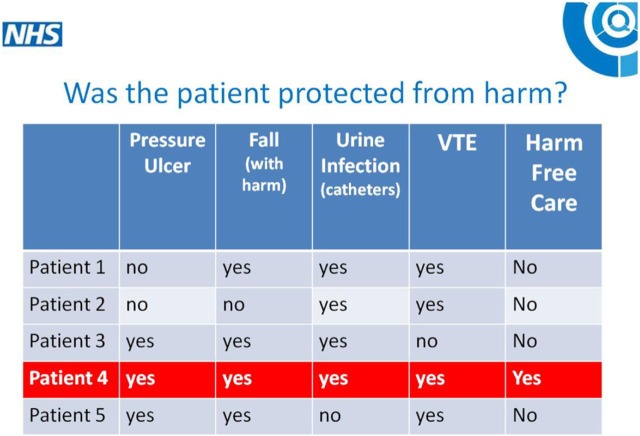
Illustration of the calculation of the harm-free care indicator. Only one patient (patient four) received “harm-free care” defined as the absence of harm from any of the four outcomes

In designing the NHS ST we have attached importance to looking at these four harms collectively because, we propose that not only are these issues biologically interdependent (often affecting the same group of elderly patients), but they are also interdependent from an improvement perspective. This theory comes from subjective report from the 2009 national VTE reduction programme which suggests that increased use of compression stockings (while appropriate and necessary for VTE prevention in some high-risk patients) can be associated with increased risk of pressure ulcers (to the heel and back of the knee).^39^ We propose that the only way to mitigate this interdependence and protect the most vulnerable is to measure VTE and pressure ulcers together in the same patient at the same time. To our knowledge, the NHS ST is unique in recognising that the patient safety improvement community could move faster and more efficiently by addressing the interdependencies between common harms (Figure 3) and designing interventions and measurement systems that mitigate the risks highlighted above.

## Conclusion

In this paper, we have been able to share important learning from the development, pilot and early testing of the NHS ST. The question over how the incentive scheme will affect the data collection (either positively or negatively) requires further investigation, in particular in the light of learning from the Matching Michigan programme which demonstrates the complexity of implementing new measurement systems in clinical settings.^38^ Over the next 6 months the focus will move away from the establishment of the measurement systems to the use of the data for improvement. This is where the focus will shift from “measuring” to measuring for improvement. The utility of the operational definitions and the data will need to be understood in the context of locally collected patient safety data and application of systematic change method will need to be considered to begin to reduce the harms identified in the NHS ST. Further publications which describe the data interpretation, use and response to financial incentives and learning from large-scale implementation of a harm measure are required to fully exploit the opportunity which this data collection affords to the global learning on harm, measurement and use of incentives.
